# Beyond Nafion with Fluorine-Free sPSU–sNIM Membranes: Nanostructured Proton Pathways for Harsh Fuel Cell Environments

**DOI:** 10.1021/acsami.5c13417

**Published:** 2025-10-16

**Authors:** Cataldo Simari, Ernestino Lufrano, Luigi Coppola, Isabella Nicotera

**Affiliations:** † Department of Chemistry and Chemical Technology, 18950 University of Calabria, Via P. Bucci, Rende, Cosenza 87036, Italy; ‡ lpm-Laboratorio Preparazione Materiali, Star-Lab, University of Calabria, Via Tito Flavio, Rende, Cosenza 87036, Italy

**Keywords:** fluorine-free membranes, sulfonated nanoscale ionic materials, sulfonated polysulfone, Grotthuss mechanism, high-temperature PEMFC, low-humidity operation, DHFC

## Abstract

In this study, nanocomposite membranes were developed by incorporating sulfonated nanoscale ionic materials (sNIMs) into a sulfonated polysulfone (sPSU) matrix for high-temperature polymer electrolyte membrane fuel cells (PEMFCs), with a focus on direct hydrogen fuel cell (DHFC) applications. The sNIMs, composed of silica nanoparticles functionalized with tethered sulfonic acid groups, were uniformly dispersed within the polymer matrix via solution casting. Structural and electrochemical characterizations demonstrated that the resulting membranes exhibit improved thermal and mechanical stability, enhanced hydration retention, and superior proton conductivity compared to pristine sPSU and recast Nafion. Remarkably, the optimized sNIM-3 formulation achieved 18  mS cm^–1^ conductivity at 120 °C and 30% RH, outperforming Nafion under identical conditions. Diffusion NMR and impedance spectroscopy revealed that the nanostructured ionic domains introduced by the sNIMs enable efficient proton transport predominantly via a Grotthuss-type hopping mechanism, even at low humidity and elevated temperatures. Fuel cell tests confirmed the exceptional performance of sNIM-3, making these membranes highly attractive fluorine-free candidates for next-generation PEMFCs.

## Graphical abstract

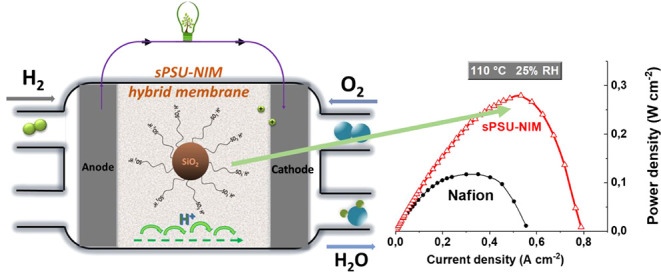

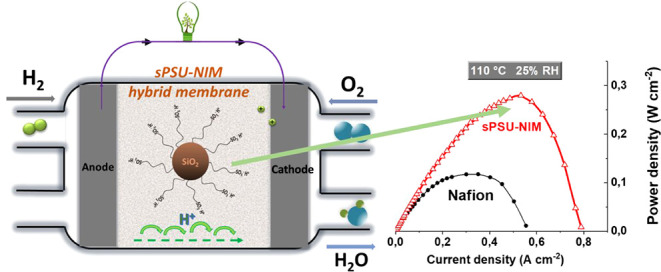

## 1 Introduction

Polymer Electrolyte Membrane Fuel Cells (PEMFCs), and particularly Direct Hydrogen Fuel Cells (DHFCs) which directly use hydrogen as fuel without requiring reforming processes, are widely regarded as promising technologies for clean and efficient energy conversion.^1–3^ However, their commercial deployment remains hindered by persistent challenges, especially when operating at elevated temperatures and under low-humidity conditions.^4^

To date, Nafion and other perfluorinated sulfonic acid (PFSA) membranes have dominated the PEM landscape due to their outstanding proton conductivity in fully hydrated environments.^5,6^ Yet, these materials exhibit significant performance degradation when humidity drops, a condition frequently encountered above 100 °C.^7,8^ More critically, their environmental persistence, cost, and reliance on fluorinated chemistry pose growing concerns. The need to transition toward *fluorine-free* alternatives is now imperative, not only from a sustainability perspective but also to reduce costs and develop scalable, eco-friendly fuel cell technologies.^9–11^

This transition, however, requires materials that can match or exceed Nafion’s benchmark in terms of chemical and mechanical stability, conductivity, and durability in real-world operating conditions. In particular, achieving stable operation above 100 °C remains a crucial target, as elevated temperatures offer key advantages for DHFCs: improved electrode kinetics, enhanced CO tolerance, and more efficient water and thermal management.^12^

Recent advances in fluorine-free polymer electrolyte membranes have shown significant promise through various approaches. Functionalized clays have emerged as effective nanofillers, with sulfonated montmorillonite demonstrating enhanced proton conductivity and improved membrane durability.^13,14^ Metal–organic frameworks (MOFs) have also gained attention as multifunctional fillers, offering both proton conduction pathways and structural reinforcement.^15–17^ Graphene oxide and its derivatives represent another promising class of nanomaterials, providing excellent mechanical properties and additional proton transport channels through surface functionalization.^18,19^ These approaches highlight the importance of nanostructured design in achieving efficient proton conduction beyond the limitations of conventional vehicular transport mechanisms.

In this context, aromatic engineering polymers such as polysulfone (PSU) represent a compelling platform. PSU offers excellent thermal and chemical stability, is cost-effective, and is inherently suitable for functional modification. Upon sulfonation, PSU (sPSU) can serve as a proton-conducting polymer, but a high degree of sulfonation is typically required to reach adequate conductivity levels, often at the expense of dimensional and mechanical stability due to excessive water uptake.^20–22^

To overcome this conductivity–stability trade-off, hybrid membrane strategies incorporating nanostructured fillers have emerged as a powerful approach.^23,24^ Among these, nanoscale ionic materials (NIMs) represent a particularly promising class. These hybrid nanoparticles consist of an inorganic core covalently grafted with a charged oligomeric corona, synthesized through a single-step process.^25^ Their structural tenability, through variation of core size, canopy architecture, and surface functionality, enables precise control over their physical and interfacial properties.^26^ Moreover, their response to external stimuli (e.g., temperature, electric fields) adds further versatility. Compared to other nanofiller approaches, sNIMs offer several unique advantages. Unlike functionalized clays which can suffer from limited dispersion and potential leaching of functional groups, sNIMs maintain exceptional colloidal stability through their covalently tethered ionic corona. While MOFs provide excellent porosity and surface area, they may face stability issues under harsh fuel cell conditions. Graphene oxide derivatives, although providing excellent mechanical reinforcement, often require complex functionalization procedures and may introduce electrical conductivity concerns. The sNIM approach combines the benefits of controlled nanostructure, chemical stability, and ease of processing while avoiding many of the limitations associated with other nanofillers.

In proton-conducting membranes, sulfonated NIMs (sNIMs) serve a dual role: they provide fixed acidic sites for proton conduction and form discrete hydrophilic domains that enhance water retention even under low humidity. Their covalent structure prevents functional group leaching and ensures both thermal and morphological stability over prolonged operation. Unlike conventional inorganic fillers, NIMs maintain exceptional colloidal stability and resist aggregation at the nanoscale. The tethered nature of the ionic corona also avoids the use of volatile dispersants, preventing evaporation-related degradation at high temperatures. In previous studies, we have successfully employed this type of nanofiller in Nafion-based systems,^27^ as well as in anion exchange membranes (AEMs) functionalized with cationic groups,^28^ demonstrating their versatility and effectiveness across a range of membrane architectures.

In this study, we explore sulfonated polysulfone (sPSU)-based hybrid membranes incorporating sNIMs as advanced, *fluorine-free* electrolytes for DHFCs. Our goal is to leverage the synergistic interactions between the polymer matrix and the sNIMs to improve hydration stability and proton transport under demanding conditions. Structural, thermal, mechanical, and electrochemical characterizations are presented, with a particular focus on membrane performance under simulated fuel cell operating conditions, including high-temperature and low-humidity environments.

## 2 Experimental Section

### 2.1 Synthesis of Sulfonated NIMs (sNIMs)

Sulfonated NIMs (sNIMs), composed of silica nanoparticles functionalized with sulfonic acid groups, were synthesized in-house following previously reported procedures^27^ and briefly described here. In a typical procedure (drafted in Scheme 1), 3 g of colloidal silica (Ludox HS 30, Sigma-Aldrich) was dispersed in 22 mL of distilled water. Separately, 4 g of 3-(trihydroxysilyl)-1-propanesulfonic acid (40 wt %, Gelest)—was diluted in 20 mL of water. The silica dispersion was then combined with the silane solution. The pH was adjusted to 5 using 1 M HCl or NaOH (Sigma-Aldrich), and the mixture was sealed and heated at 70 °C for 24 h under continuous stirring. After reaction, the suspension was transferred into dialysis tubing (MWCO 10 kDa, 32 mm width, Sigma-Aldrich) and dialyzed for at least 72 h. The resulting sNIMs were purified by dialysis and ion-exchange treatment to remove residual sodium ions and unreacted species.

**Scheme 1:**
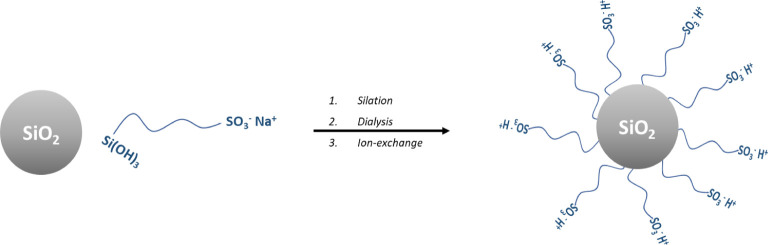
Schematic Illustration of the Functionalized sNIM Nanoparticles Synthesis

### 2.2 Synthesis of Sulfonated Polysulfone (sPSU)

The sulfonation of PSU was carried out following a controlled protocol using trimethylsilyl chlorosulfonate as the sulfonating agent.^29^ Dried PSU was dissolved in anhydrous chloroform at room temperature, and the sulfonating agent was added in a molar ratio of 2.5:1 (agent:repeating unit). The reaction was conducted under reflux at 50 °C for 6 h. After sulfonation, the resulting silyl sulfonated polymer was converted into its sodium salt form via treatment with sodium methoxide solution, followed by precipitation in ethanol. The precipitate was filtered, washed thoroughly with ethanol and deionized water, and dried at 60 °C.

### 2.3 Membrane Fabrication

To prepare pristine membranes, sPSU was dissolved in DMAc (10 wt %) at room temperature until a clear solution was obtained. For nanocomposites, the appropriate amount of sNIMs (3, 5, and 10 wt % with respect to polymer weight) was dispersed in DMAc via ultrasonication and stirring for 24 h. The sNIM dispersion was added to the sPSU solution and stirred further to ensure homogeneity. The solutions were cast onto Petri dishes and dried at 60 °C. Membranes were then converted to the acid form by immersion in 1 M H_2_SO_4_ at 60 °C for 6 h, followed by thorough rinsing with deionized boiling water.

Scanning Electron Microscopy (SEM, Cambridge Stereoscan 360, 15 kV, cryo-fractured samples) revealed that the cross sections of both pristine and nanocomposite membranes are dense, smooth, and free of defects. In particular, the nanocomposite membrane shows a homogeneous nanodispersion of the filler particles without any nanometric agglomeration, indicating a uniform distribution of sNIMs within the polymer matrix (see Figure S1).

### 2.4 Characterization Techniques

The ion exchange capacity (IEC) of the electrolyte membranes was determined by conventional acid–base titration. Protonated membranes were first dried in a vacuum oven at 60 °C for 24 h until a constant dry weight (*m*_dry_) was reached, measured using an analytical balance (Radvag, Model AS 220.R2 Plus, ±0.0001 g). Each dried membrane was then immersed in 50 mL of 1.0 M NaCl solution for 24 h to enable complete proton-to-sodium ion exchange. The resulting acidic solution was titrated with 0.01 M NaOH using phenolphthalein as the indicator. All titrations were performed in triplicate, and the IEC (meq g^–1^) was calculated using the following equation (eq 1):

1 $$\mathrm{IEC}=\frac{M_{\left(\mathrm{NaOH}\right)}V_{\left(\mathrm{NaOH}\right)}}{W_{\mathrm{dry}}};\;\mathrm{meq}\;g^{-1}$$

The water uptake (w.u.) was determined at room temperature (∼25 °C). Prior to measurement, pretreated membranes were thoroughly dried in a vacuum oven at 60 °C for 24 h to obtain a constant dry weight (*m*_dry_). The dried membranes were then immersed in deionized water at room temperature for 24 h to achieve equilibrium swelling. After soaking, surface water was gently removed using laboratory wipes, and the swollen membranes were immediately weighed (*m*_wet_). The water uptake was calculated as

2 $$\mathrm{w.u.}=\frac{m_{\mathrm{wet}}-m_{\mathrm{dry}}}{m_{\mathrm{dry}}}-100;\;\%$$

All measurements were repeated three times, and results are reported as mean ± standard deviation.

Thermogravimetric analysis (TGA) was performed using a Netzsch STA 449C Jupiter thermal analyzer (Netzsch-Gerätebau GmbH, Selb, Germany). Samples were heated in alumina crucibles from 25 to 700 °C at a rate of 10 °C/min under a nitrogen flow of 16 cm^3^ min^–1^.

Dynamic mechanical analysis (DMA) was carried out using a Metravib DMA/25 instrument equipped with shear clamps. Rectangular membrane strips were tested under a sinusoidal stress of 10^–3^ Pa at 1 Hz, while being heated from 25 to 250 °C at a rate of 3 °C/min. All measurements were conducted on dry samples in ambient atmosphere (i.e., without humidity control).

The self-diffusion coefficient (*D*) of water within the polymer electrolyte membranes (PEMs) was determined using Pulsed Field Gradient Nuclear Magnetic Resonance (PFG NMR) spectroscopy. NMR experiments were conducted on a Bruker AVANCE 300 wide bore spectrometer operating at a proton resonance frequency of 300 MHz, which was outfitted with a Diff30 Z-diffusion 30 G/cm/A multinuclear probe with substitutable RF inserts and capable of generating pulsed field gradients up to 1200 G/cm. Prior to measurement, the samples were equilibrated in deionized water at 25 °C for 24 h to achieve equilibrium water uptake. Following equilibration, the samples were carefully transferred into standard 5 mm NMR tubes, with meticulous attention paid to minimizing any water loss during this transfer.

The Pulsed Gradient Stimulated Echo (PGSTE) sequence was employed for all diffusion measurements, a sequence selected due to its superior performance with samples exhibiting shorter T2 relaxation times, a common characteristic of water in polymeric environments.

The underlying principle for signal attenuation, *I*(*g*), as a function of the gradient strength (*g*), is comprehensively described by the Stejskal–Tanner equation:^30^

3 $$I=I_{0}\;e^{-[{\left(\gamma g\delta \right)}^{2}\left(\Delta -\frac{\delta }{3}\right)]D}$$

where *I*_0_ represents the signal intensity in the absence of field gradients, γ is the gyromagnetic ratio for ^1^H, δ denotes the duration of the gradient pulses, and Δ the diffusion time.

Typical experimental parameters included a gradient pulse duration (δ) of 1 ms, a diffusion time (Δ) of 10 ms, and the application of 10 linearly spaced gradient increments, while the maximum gradient strength reached 850 G cm^–1^. A total of 16 scans were accumulated per data point to ensure an adequate signal-to-noise ratio, with a recycle delay of 2 s utilized to allow for complete spin relaxation. *D* was subsequently extracted by accurately fitting the acquired signal attenuation data to the Stejskal-Tanner equation. In all the cases, a single exponential decay was observed, indicating a relatively homogeneous diffusion environment for water within the membrane on the NMR time scale. Diffusivity was investigating in the range 20–130 °C, with steps of 20 °C and 15 min of equilibration time for each temperature.

The in-plane proton conductivity (σ) of the prepared membranes was determined using Electrochemical Impedance Spectroscopy (EIS). Measurements were performed on rectangular membrane samples (2.5 cm × 1.0 cm) with a commercial four-electrode conductivity cell (BT-112, Scribner Associates Inc.) coupled to a PGSTAT 30 potentiostat/galvanostat (Methrom Autolab) equipped with an FRA module. Measurements were performed over a range of relevant temperatures (e.g., from 30 to 120 °C in 30 °C increments) and relative humidities (RH) (e.g., from 30% RH to 90% RH). A humidification system (Fuel Cells Technologies, Inc.) was directly connected to the cell to control temperature and relative humidity (RH) through precise control of the incoming gas streams (humidified air). For each condition, the membrane was allowed to equilibrate for at least 30 min before impedance data acquisition. The AC impedance response of the cell was then measured over a frequency range of 1 Hz to 1 MHz, with a perturbation amplitude of 10 mV at open circuit potential. The membrane resistance (*R*) was determined from the high-frequency intercept of the Nyquist plot with the real impedance axis (*Z*′). The proton conductivity (σ) was calculated using the following equation:

4 $$\sigma =\frac{d}{R\times A};\;{\mathrm{S cm}}^{-1}$$

where *d* is the distance between the electrodes and *A* is the active surface area.

The electrochemical performance of the prepared polymer electrolyte membranes was evaluated in a single H_2_/O_2_ fuel cell configuration, using a commercial fuel cell test station (850C, Scribner Associates Inc.). The test station was equipped with a 5 cm^2^ single cell hardware, featuring parallel flow channels and graphite bipolar plates. For catalyst preparation, a commercial platinum-on-carbon catalyst (40% Pt/C, Alfa Aesar) was thoroughly dispersed with 33% Nafion ionomer (from a 20% ionomer solution, Ion Power) via sonication. This well-dispersed catalytic ink was subsequently deposited onto the backing layer of a Sigracet 25-BC Gas Diffusion Layer (SGL). The platinum loading on both the anode and cathode was set at 0.5 mg cm^– 2^. Membrane electrode assemblies (MEAs) were fabricated by hot-pressing the electrodes onto the prepared PEMs on both the anode and cathode at 130 °C and 30 kg cm^–2^ for 2 min. The fuel cell tests were conducted were carried out via galvanostatic measurements under controlled conditions, including a cell temperature of 80 and 110 °C and RH varying from 100 until 25%. High-purity hydrogen (99.999% H_2_) and oxygen (99.999% O_2_) were supplied to the anode and cathode, respectively. The gas flow rates were precisely controlled by mass flow controllers to ensure constant stoichiometric ratios; specifically, a stoichiometry of 1.5 for hydrogen at the anode and 2.0 for oxygen at the cathode was maintained throughout the experiments, based on the current drawn from the cell.

## 3 Results and Discussion

### 3.1 Thermal Properties and Mechanical Stability

The integration of sNIM nanoparticles into the sPSU matrix was evaluated in terms of thermal and mechanical stability, key factors for ensuring membrane durability during fuel cell operation. Thermogravimetric analysis (Figure 1a) highlights clear differences between the unfilled sPSU and the nanocomposite membranes over the 400–750 °C range. In the case of sPSU, two main weight-loss events are observed: the first, between 200 and 400 °C, is attributed to the degradation of sulfonic acid groups, while the second, occurring above 400 °C, corresponds to the breakdown of the aromatic polymer backbone.^31^

**Figure 1:**
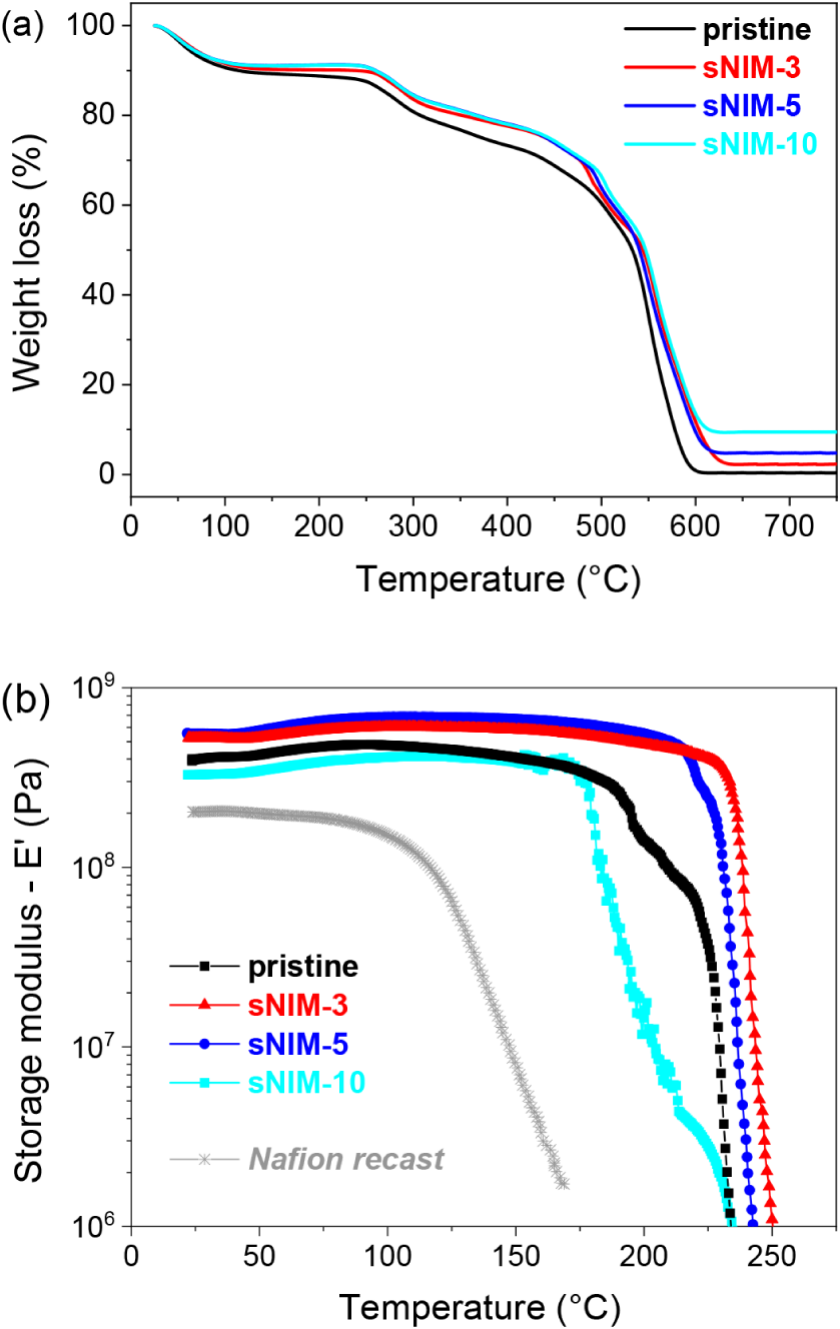
(a) Thermogravimetric analysis of the pristine sPSU and sNIM-based hybrid membranes; (b) storage modulus as a function of the temperature (Nafion recast benchmark is also reported).

Upon incorporation of sNIMs, both degradation steps shift to higher temperatures, indicating a substantial enhancement in thermal resistance due to the presence of the nanofiller. Furthermore, the amount of residual mass at 700 °C increases with the filler content, consistent with the presence of thermally stable silica structures. This residual mass not only confirms the filler’s presence but also supports the hypothesis of homogeneous dispersion within the polymer phase. Overall, the thermal behavior of the composite membranes reflects the stabilizing effect of the sNIMs and suggests an effective interaction between filler and matrix.

Dynamic Mechanical Analysis (DMA) was carried out on both pristine sPSU and sPSU-based hybrid membranes containing sNIMs, with Nafion recast included as a benchmark. The temperature-dependent storage modulus (*E*′), recorded in the range of 20–280 °C, is shown in Figure 1b. All membranes exhibited excellent mechanical stability, maintaining structural integrity without signs of deformation or physical degradation across the tested range.

A single, broad relaxation peak (α-transition), associated with the segmental motion of ionic domains, was observed for all sulfonated samples. In the hybrid membranes, this peak shifted to higher temperatures indicating improved resistance to thermally induced softening. Notably, the optimal formulation (sNIM-3) displayed this transition above 240 °C, suggesting enhanced thermo-mechanical robustness compared to the pristine sPSU, which began to soften around 200 °C.

Although a slight decrease in stiffness was observed at higher filler loadings, the overall mechanical performance of the nanocomposites remained superior to that of the unfilled membrane. Compared to Nafion, which exhibits an α-transition near 120 °C,^32,33^ the sPSU-based systems demonstrated significantly greater thermal stability. This broader resistance range positions sPSU-sNIM membranes as promising candidates for high-temperature fuel cell operation, where materials must withstand prolonged thermal and mechanical stress beyond the capabilities of conventional perfluorinated membranes.

### 3.2 Hydration Behavior and Proton Transport Properties

Table 1 reports the water uptake and ion exchange capacity (IEC) of sulfonated polysulfone (sPSU) membranes, including the pristine polymer and hybrid membranes incorporating increasing amounts (3–10 wt %) of sulfonated NIM filler. Compared to the pristine membrane (26% water uptake and 1.36 mequiv/g IEC), the introduction of NIM leads to a gradual enhancement of both properties, with the sNIM-5 membrane showing the highest values (30% and 1.52 mequiv/g, respectively). This suggests that the addition of sulfonated NIM improves membrane hydrophilicity and the density of ion-exchange sites. However, at higher loading (10 wt %), a slight decrease in both parameters is observed, likely due to filler aggregation or reduced dispersion, which may limit its interaction with the polymer matrix.

**Table 1:** Exchange Capacity and Water Uptake (after Equilibration by Soaking in DI Water at 25 °C) at Different Filler Loadings in the sPSU–sNIM Hybrid Membranes

Membranes	Water uptake (%wt ± 1)	IEC (meq/gr)
sPSU pristine	26	1.36
sNIM-3	29	1.49
sNIM-5	30	1.52
sNIM-10	28	1.39

The nanoconfined water dynamics in the membranes were studied through temperature-dependent self-diffusion coefficients measured by ^1^H PFG-NMR spectroscopy, covering a broad temperature range from room temperature to 130 °C (Figure 2). In the low-temperature regime (20–60 °C), all sPSU-based membranes exhibited similar diffusivity values, consistent with their comparable water uptake. However, at higher temperatures, distinct differences emerged, clearly reflecting the influence of both membrane composition and internal structure.

**Figure 2:**
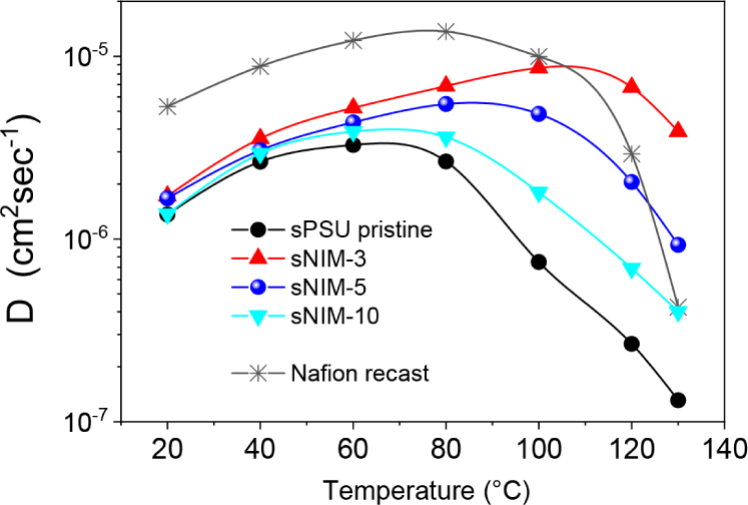
Temperature-dependent self-diffusion coefficients measured by ^1^H PFG-NMR spectroscopy, from room temperature to 130 °C.

Pristine sPSU showed a continuous decline in water diffusivity with increasing temperature, reaching values nearly 1 order of magnitude lower at 130 °C. This behavior is attributed to the progressive loss of bulk-like water, particularly the fraction weakly bound to the polymer. In contrast, hybrid membranes incorporating sulfonated NIMs demonstrated a slower decay in *D*, indicating improved water retention. This effect is particularly pronounced in the sNIM-3 membrane, where diffusivity increases with temperature up to 120 °C and remains nearly constant thereafter. Such behavior suggests not only enhanced hydration stability but also the activation of a more efficient transport mechanism.

This remarkable performance can be ascribed to the high density of sulfonic acid groups decorating the NIM surface, which enhances the membrane’s ability to retain water even at elevated temperatures, without the need for external humidification. Furthermore, the anomalous increase in *D* observed for the sNIM-3 sample strongly suggests the emergence of a Grotthuss-like mechanism, in which proton hopping between hydrogen-bonded water molecules becomes dominant over vehicular transport. This is further supported by the high proton diffusivity sustained at 120–130 °C, far exceeding that of both pristine PSU and the other hybrid membranes.

The behavior of a recast Nafion membrane was also analyzed for comparison. As expected, Nafion exhibits higher water mobility, which can be attributed to its less branched morphology compared to aromatic polymer-based membranes, and to the presence of large pores and well-developed hydrophilic channels that offer lower tortuosity to water transport.^34^ However, above 80 °C, the rapid evaporation of bulk water causes a sharp drop in diffusivity and a corresponding loss of proton conductivity. Conversely, the hybrid PSU-based membranes, particularly sNIM-3, maintain significant proton mobility in this temperature range, thanks to the cooperative effect of nanoparticle functionalization and nanostructure-driven water stabilization.

The impact of sulfonated NIM nanoparticles on proton transport was further assessed by electrochemical impedance spectroscopy (EIS), performed under four relative humidity (RH) conditions across the temperature range of 30–120 °C (Figure 3). For clarity, different *y*-axis scales were adopted in panels a–b and c–d to better visualize the differences among the membranes. The conductivity trends are fully consistent with the PFG-NMR findings, confirming that a filler loading of 3 wt% represents the optimal condition to induce a nanostructure capable of supporting efficient proton mobility.

**Figure 3:**
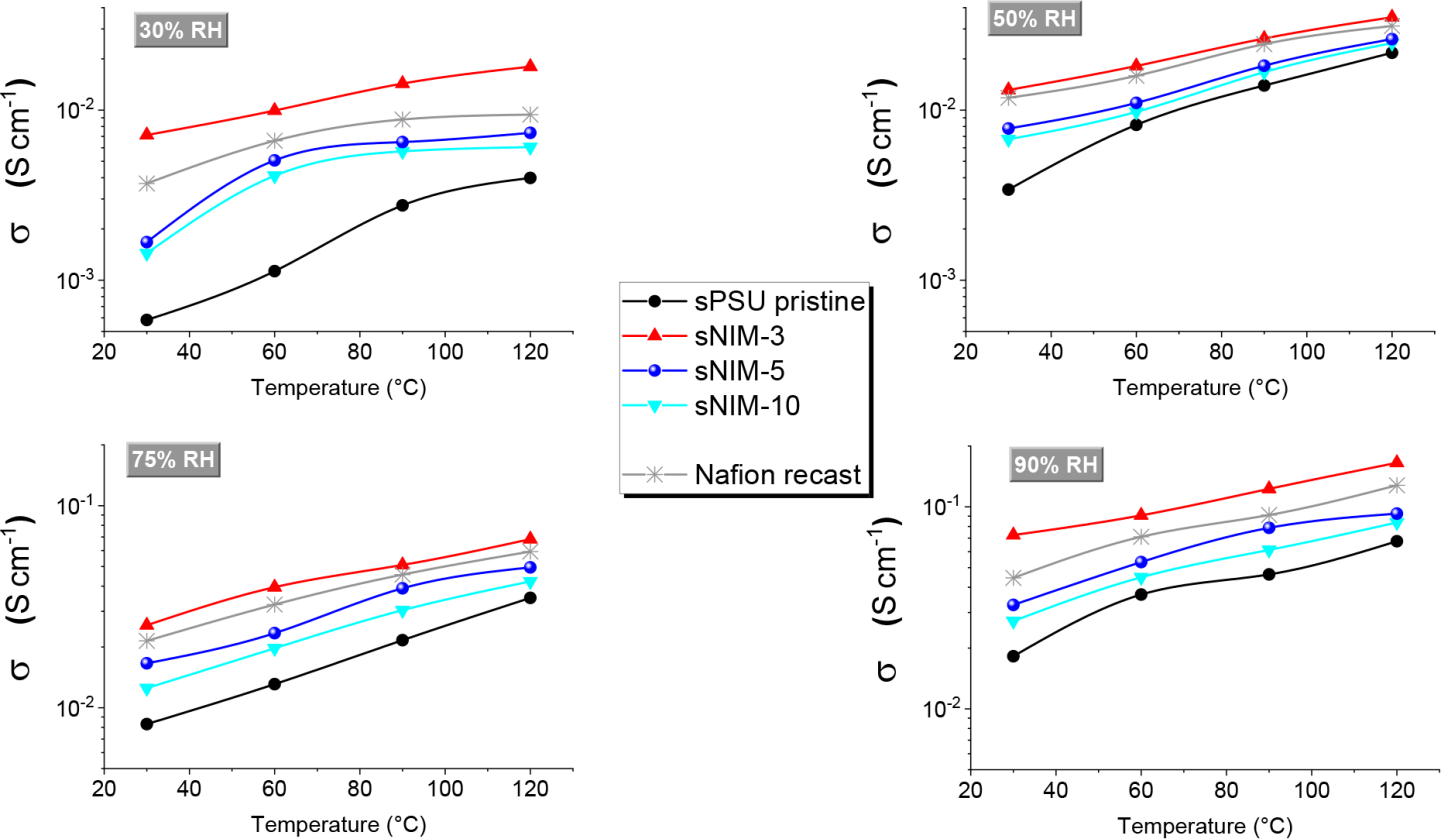
Proton conductivities measured by EIS by in-plane configuration, at different RH% (30, 50 and 90%) and in the *T*-range 30–120 °C. The σ values of recast Nafion has been reported as benchmark.

Indeed, the sPSU-based membrane containing 3 wt% sNIM outperforms both the pristine polymer and the other hybrid membranes with 5% and 10% filler content, which exhibit significantly lower proton conductivity under all tested conditions. The recast Nafion membrane, included as a benchmark, also shows inferior performance, particularly at elevated temperatures and low humidity. At 120 °C and 30% RH, the 3% sNIM membrane reaches a conductivity of 18 mS cm^–1^, compared to 9 mS cm^–1^ for Nafion, highlighting the excellent high-temperature and low-humidity conductivity of the optimized composite.

This enhancement is particularly remarkable considering the inherent limitations of hydrocarbon-based polymers. Unlike Nafion, sPSU lacks strong microphase separation and exhibits weaker acidity, which typically leads to narrower, less connected hydrophilic domains, factors that limit proton mobility.^35^ However, the uniform dispersion of highly sulfonated NIM nanoparticles within the sPSU matrix introduces both additional fixed ionic sites and hydrophilic domains. This dual functionality not only improves water retention at high temperature, as evidenced by NMR data, but also facilitates the formation of well-connected proton pathways. The resulting morphology supports a dominant Grotthuss-type transport mechanism, allowing efficient proton hopping through hydrogen-bonded water networks.

These results emphasize that the effectiveness of a nanocomposite membrane is governed not only by the chemical nature of the polymer and the filler, but also, critically, by the nanoscale architecture, the quality of dispersion, and the specific interactions established within the hybrid structure. The sNIM-3 membrane stands as a clear demonstration of how rational nanostructuring can overcome the intrinsic limitations of hydrocarbon-based electrolytes, unlocking high proton conductivity even under demanding operating conditions.

In addition, EIS measurements were performed in both in-plane and through-plane configurations to assess potential anisotropy in proton transport. While the in-plane results are reported in Figures 3, the Figure 4 shows a direct comparison between the two configurations at the maximum humidification (90% RH) for all membranes. The nearly identical conductivity values obtained in both directions clearly demonstrate the absence of spatial anisotropy in the membranes confirming that proton transport occurs uniformly across the membrane plane and thickness. This isotropic behavior indicates a homogeneous distribution of ionic pathways throughout the membrane thickness, which is a key indicator of uniform nanostructuring. This observation further validates the homogeneous nanostructure achieved through the fine dispersion of sNIM nanoparticles within the sPSU matrix.

**Figure 4:**
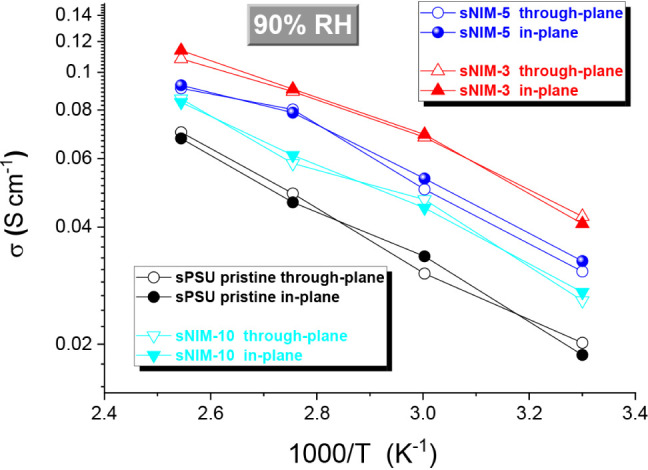
Arrhenius plots of proton conductivities measured by both in-plane and through-plane configurations, at different 90% RH.

### 3.3 H_2_/O_2_ Fuel Cell Performances and Durability

Figure 5 presents the polarization and power density curves of H_2_/O_2_ single PEM fuel cells assembled with Nafion recast, pristine sPSU, and sPSU-based hybrid membranes incorporating sulfonated sNIM fillers (sNIM-3 and sNIM-5). The tests were carried out under progressively harsher operating conditions, ranging from 80 °C at full humidification (100% RH) to 110 °C with only 25% RH. This testing strategy reflects a growing trend in PEMFC research, which is moving beyond conventional mild conditions, typically below 100 °C and high humidity, toward more application-relevant environments. The present study aligns with this shift by assessing membrane performance under stringent thermal and hydration stress, thus providing insights into their suitability for real-world fuel cell operation.^3,36^

**Figure 5:**
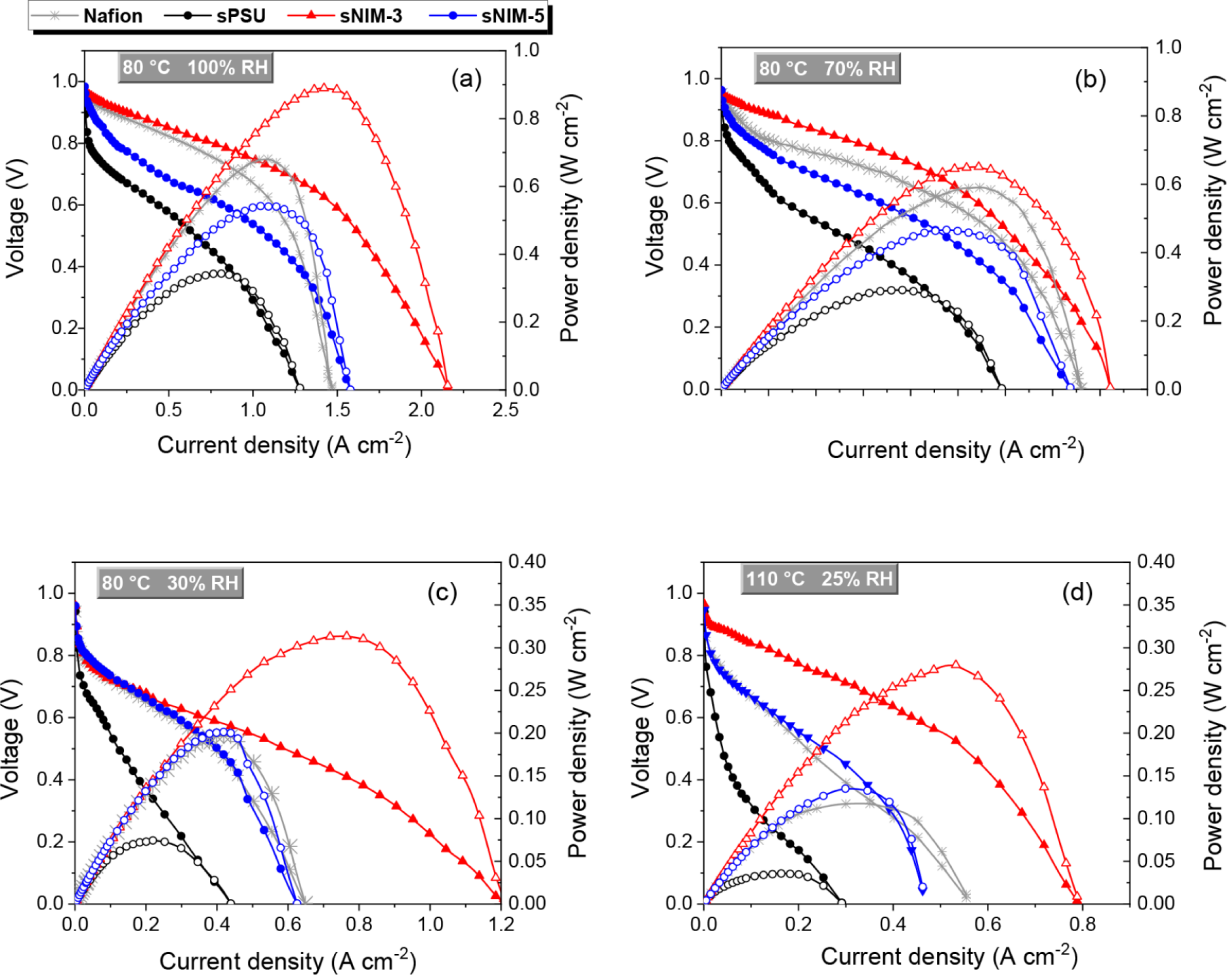
Polarization (filled symbols) and power curves (open symbols) of H_2_/O_2_ PEMFCs at (a) 80 °C/100% RH, (b) 80 °C/70% RH, (c) 80 °C/30% RH and (d) 110 °C/25% RH. The flux rates of H_2_ and O_2_ where both 200 mL min^–1^.

At 80 °C and 100% RH, a condition where Nafion typically achieves its maximum performance, the sNIM-3 membrane delivers a significantly higher power density. This is a remarkable result, considering that sNIM-3 is a nonfluorinated, hydrocarbon-based membrane. Its superior output under fully humidified conditions highlights the efficacy of the sulfonated NIM additive in facilitating proton conduction and water management. The voltage profile of sNIM-3 is also notably more stable across the current density range, indicating reduced activation and ohmic losses compared to Nafion. Upon reducing the relative humidity to 70%, performance declines for all membranes, yet sNIM-3 remains the top performer. At 30% RH, the performance gap between the membranes becomes more pronounced. While Nafion and sNIM-5 exhibit comparable behavior, sNIM-3 stands out with a significantly higher peak power density and open-circuit voltage, reflecting improved membrane hydration and lower gas crossover.

Under the most challenging condition of 110 °C and 25% RH, Nafion and sNIM-5 both experience sharp performance drops, with overlapping current–voltage characteristics. In contrast, sNIM-3 maintains a relatively high output, delivering a power density well above the other membranes. This result underscores the robustness of the sNIM-3 membrane, whose nanostructured sulfonated filler appears to play a critical role in preserving membrane hydration and proton transport even in extremely dry environments.

Overall, sNIM-3 exhibits consistently superior performance across all conditions, surpassing Nafion even under full humidification, and maintaining clear advantages at reduced RH and elevated temperature. These results highlight the potential of sulfonated NIM-based sPSU membranes as promising, nonfluorinated alternatives for advanced PEMFCs.

The main electrochemical parameters, including open circuit voltage (OCV), maximum current density, and peak power density, were measured for all samples and are summarized in Table 2. Additionally, the potential and current density values at 0.6 V are reported in Tables S1 and S2.

**Table 2:**
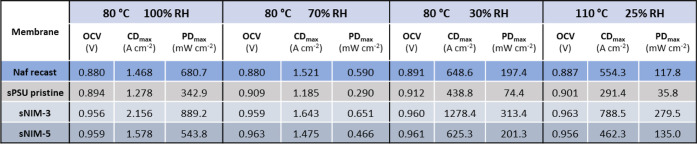
Electrochemical Parameters (Current Density = CD and Power Density = PD) of MEAs Assembled with Different Membranes, under Different Operating Conditions

The incorporation of sulfonated NIM (sNIM) nanoparticles into the sPSU matrix enhances the OCV, indicating a reduction in gas permeability through the membrane. Among the tested membranes, the MEA based on the sNIM-5 sample exhibited the highest OCV (0.959 V), outperforming both recast Nafion (0.880 V) and pristine sPSU (0.894 V).

Remarkably, the sNIM-3 membrane achieved a peak power density of 279.5 mW cm^–2^ at 110 °C and 25% relative humidity (RH), which is nearly 2.5 times higher than that of Nafion under the same conditions (117.8 mW cm^–2^). Even under more moderate operating conditions (80 °C and 100% RH), the sNIM-3 sample maintained excellent performance, reaching a peak power density of 890 mW cm^–2^, approximately 25% higher than that of Nafion (681 mW cm^–^ ). It is worth highlighting that the measured values for Nafion align well with the range commonly found in previous studies.^37,38^

In conclusion, the sPSU-based hybrid membranes not only outperform Nafion in terms of electrochemical performance but also maintain excellent functionality at elevated temperatures and reduced humidity levels. This resilience suggests an intrinsic capacity for self-hydration, highlighting their strong potential for use in next-generation fuel cell systems designed to operate beyond standard laboratory settings.

The durability of sNIM-3 membrane was finally assessed by chemical accelerated stress test (AST) accomplished by an open circuit voltage (OCV) hold at 110 °C and 25% RH (Figure 6), according to the US Department of Energy suggested protocols.^39,40^ This type of test, is designed to simulate harsh operating conditions and accelerate degradation mechanisms, particularly chemical degradation via radical attack and membrane thinning due to high temperature and low humidity. Nafion benchmark exhibits rapid and severe OCV decay in the first 10 h, followed by a progressive decline of the cell potential. This is consistent with its known poor performance in dry, hot environments where dehydration leads to catastrophic chemical and mechanical breakdown.^41^ In contrast, the sPSU membrane, despite an OCV decay rate of 3.79 mV/h, demonstrates a more extended operational lifespan before reaching critical failure, suggesting an improved, though still limited, intrinsic stability compared to Nafion under these conditions. However, the standout performer is the sNIM-3 nanocomposite, which achieves significantly superior durability with an impressively low OCV decay rate of just 1.69 mV/h. This marked improvement is directly attributable to the strategic incorporation of sulfonated nanoscale ionic materials into the sPSU matrix. As previously established, these nanoparticles are able to significantly improve the mechanical robustness of the membrane and increase its water retention at low humidity. Furthermore, recent studies have demonstrated that silica-based materials, including functionalized silica nanoparticles like sNIMs, can act as effective radical scavengers in PEMFC environments. These materials effectively neutralize highly reactive oxygen species such as hydroxyl radicals that are primary culprits in polymer backbone degradation, through mechanisms including H_2_O_2_ complexation and blocking of Fenton-like reactions. Specifically, silica nanoparticles have been shown to suppress H_2_O_2_ formation and provide prolonged membrane lifetime under accelerated stress conditions.^42–45^ These combined effects, proven enhancements in mechanical integrity and water management, alongside established radical scavenging capabilities, collectively mitigate the chemical and mechanical degradation pathways, thereby extending the operational lifespan of the sNIM-3 membrane far beyond its sPSU counterpart.

**Figure 6:**
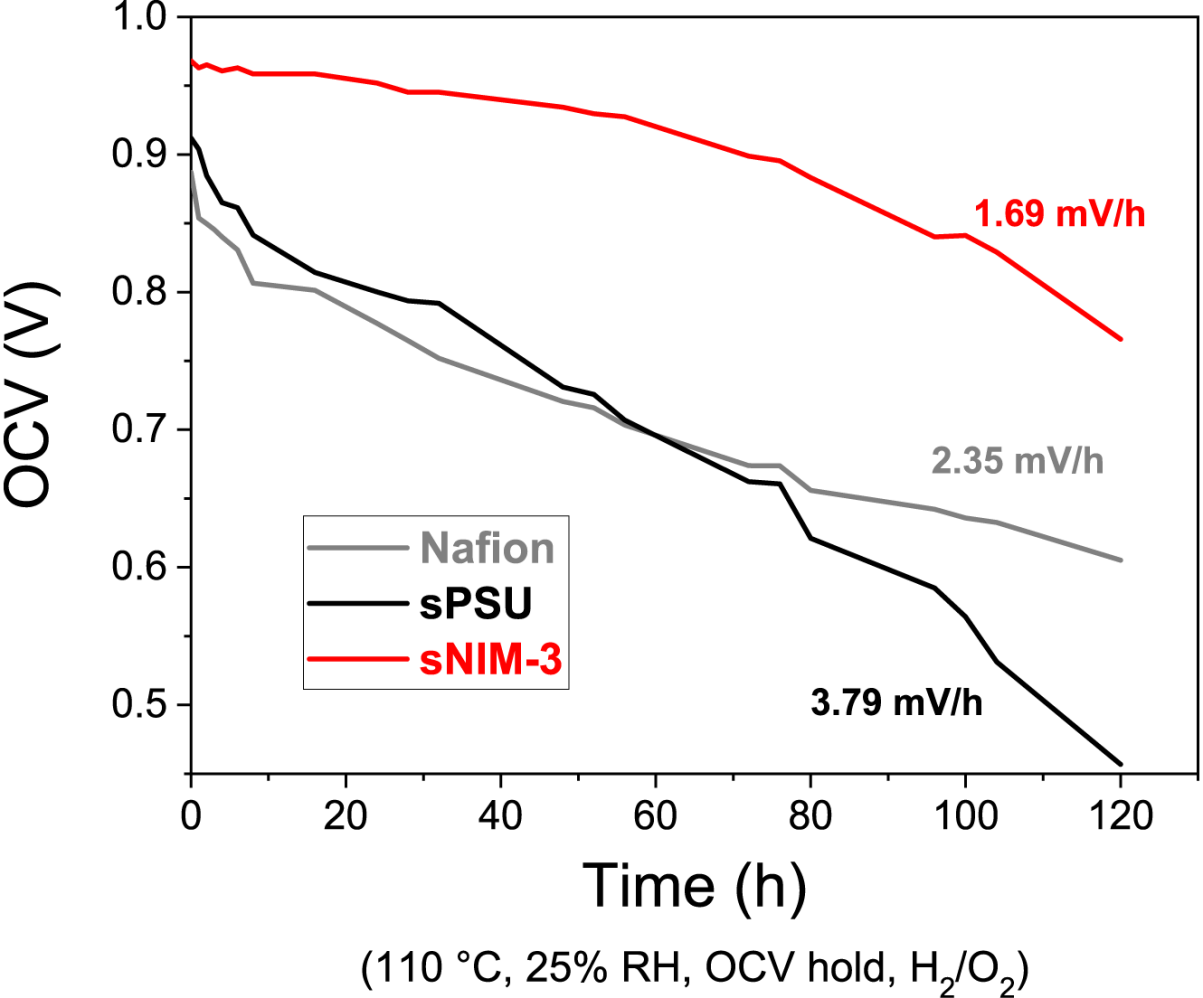
Durability under chemical accelerated stress test of an OCV hold at 110 °C and 20% RH.

## 4 Conclusions

This work presents the successful design of sulfonated polysulfone (sPSU)-based nanocomposite membranes incorporating sulfonated nanoscale ionic materials (sNIMs) for high-temperature, low-humidity operation in DHFCs. The tailored inclusion of sNIMs yields membranes with enhanced mechanical robustness, thermal stability, and hydration retention, enabled by the formation of well-dispersed, nanostructured ionic domains.

A key outcome of this study is the demonstration that sNIMs not only contribute additional proton-conducting sites but also foster the development of interconnected hydrogen-bond networks that support Grotthuss-type proton transport. This hopping mechanism, dominant at high temperatures and reduced humidity, was clearly evidenced by the anomalous increase in proton diffusivity observed via NMR and by the sustained high conductivity under dry conditions. The sNIM-3 membrane, in particular, achieved outstanding proton conductivity (18 mS cm^–1^ at 120 °C, 30% RH) and exceptional fuel cell performance, with peak power densities significantly surpassing Nafion even at minimal humidification.

These results highlight the fundamental importance of nanostructured design in achieving efficient proton conduction beyond the limitations of conventional vehicular transport. The sPSU-sNIM membranes, leveraging a Grotthuss-dominated mechanism, offer a compelling fluorine-free pathway for next-generation PEMFCs capable of durable and efficient operation in harsh conditions.

## Supplementary Material



## Notes

Tables S1 and S2: Electrochemical parameters (current density = CD and power density = PD) of MEAs assembled with different membranes, under different operating conditions; cross-sectional SEM images (Figure S1) (PDF)
