# Supplementary material for: Beyond Nafion with Fluorine-Free sPSU–sNIM Membranes: Nanostructured Proton Pathways for Harsh Fuel Cell Environments
Source: ACS Appl Mater Interfaces. 2025 Oct 16;17(43):59378–87. doi: 10.1021/acsami.5c13417 (PMC12581127; doi:10.1021/acsami.5c13417)
Supplement: Supplementary file 1 [file am5c13417_si_001.pdf]

Supporting Information

Beyond Nafion with Fluorine-Free sPSU–sNIM Membranes: Nanostructured Proton Pathways for Harsh Fuel Cell Environments

Cataldo Simari [1], Ernestino Lufrano [1], Luigi Coppola [1], Isabella Nicotera [1,2] *

[1] Department of Chemistry and Chemical Technology. University of Calabria, Via P. Bucci, Rende (CS), 87036, Italy

[2] LPM-Laboratorio Preparazione Materiali, STAR-Lab, University of Calabria, Via Tito Flavio, Rende (CS) 87036, Italy

Corresponding Author email: isabella.nicotera@unical.it

Table S1. *Electrochemical parameters (Current density=CD and Power density = PD) of MEAs assembled with different membranes, under different operating conditions*

Membrane	80 °C 30% RH					110 °C 25% RH				
	OCV (V)	CD @ 0.6V (mA cm^{-2})	PD @ 0.6V (mW cm^{-2})	CD_{max} (mA cm^{-2})	PD_{max} (mW cm^{-2})	OCV (V)	CD @ 0.6V (mA cm^{-2})	PD @ 0.6V (mW cm^{-2})	CD_{max} (mA cm^{-2})	PD_{max} (mW cm^{-2})
**Naf recast**	0.891	278.4	168.4	648.6	197.4	0.887	147.6	88.9	554.3	117.8
**sPSU**	0.912	79.9	47.4	438.8	74.4	0.901	24.0	14.6	291.4	35.8
**sNIM-3**	0.960	369.1	221.2	1278.4	313.4	0.963	438.6	264.2	788.5	279.5
**sNIM-5**	0.961	288.6	173.4	625.3	201.3	0.956	154.1	93.2	462.3	135.0

Table S2. *Electrochemical parameters (Current density=CD and Power density = PD) of MEAs assembled with different membranes, under different operating conditions*

Membrane	80 °C 70% RH					80 °C 100% RH				
	OCV (V)	CD @ 0.6V (A cm^{-2})	PD @ 0.6V (mW cm^{-2})	CD_{max} (A cm^{-2})	PD_{max} (mW cm^{-2})	OCV (V)	CD @ 0.6V (A cm^{-2})	PD @ 0.6V (mW cm^{-2})	CD_{max} (A cm^{-2})	PD_{max} (mW cm^{-2})
**Naf recast**	0.880	0.951	574.0	1.521	0.590	0.880	1.113	677.3	1.468	680.7
**sPSU**	0.909	0.276	164.4	1.185	0.290	0.894	0.416	252.7	1.278	342.9
**sNIM-3**	0.959	1.088	650.8	1.643	0.651	0.956	1.498	885.6	2.156	889.2
**sNIM-5**	0.963	0.695	413.2	1.475	0.466	0.959	0.812	489.1	1.578	543.8

Morphological Analysis

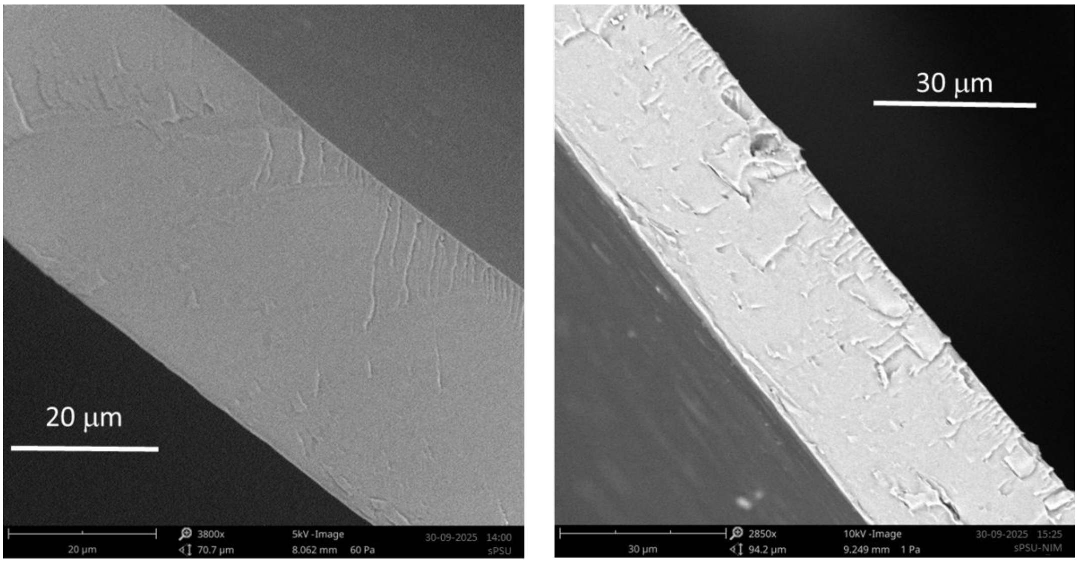

Figure S1: Cross-sectional SEM images acquired on sPSU pristine membrane (on the left) and sNIM-3 nanocomposite membrane (on the right), respectively.
